# In or out of control: Modulating regulatory T cell homeostasis and function with immune checkpoint pathways

**DOI:** 10.3389/fimmu.2022.1033705

**Published:** 2022-12-15

**Authors:** Maha Abdeladhim, Jodi L. Karnell, Sadiye Amcaoglu Rieder

**Affiliations:** Department of Research, Horizon Therapeutics, Rockville, MD, United States

**Keywords:** Treg - regulatory T cell, immune checkpoint, costimulatory pathway, coinhibition modulation, cancer, autoimmunity

## Abstract

Regulatory T cells (Tregs) are the master regulators of immunity and they have been implicated in different disease states such as infection, autoimmunity and cancer. Since their discovery, many studies have focused on understanding Treg development, differentiation, and function. While there are many players in the generation and function of truly suppressive Tregs, the role of checkpoint pathways in these processes have been studied extensively. In this paper, we systematically review the role of different checkpoint pathways in Treg homeostasis and function. We describe how co-stimulatory and co-inhibitory pathways modulate Treg homeostasis and function and highlight data from mouse and human studies. Multiple checkpoint pathways are being targeted in cancer and autoimmunity; therefore, we share insights from the clinic and discuss the effect of experimental and approved therapeutics on Treg biology.

## Introduction

The immune system employs multiple players and mechanisms to protect the host from infections, autoimmunity, and cancer. Innate and adaptive arms of the immune system collaborate to maintain homeostasis and to protect the host from foreign antigens while maintaining the distinction between foreign and self (1). One integral lymphoid population in the adaptive immune system is the T cell and broadly there are two major populations—effector T cells (Teffs, including helper, cytotoxic, memory and γδ subsets) and regulatory T cells (Tregs). While Teffs are necessary to mount an effective immune response against foreign antigens, Tregs are essential for maintaining tolerance to self (2). Like all T cells, Tregs are developed and trained in the thymus (3). T cells with high affinity to self-antigens are deleted during negative selection while cells with intermediate affinity to self-antigens develop into Tregs. Due to imperfections of the negative selection process, Teffs with high affinity TCRs for self-antigens are sometimes released into the periphery (4). One of the main functions of Tregs is to control these self-reactive cells and maintain peripheral tolerance (5).

**Figure 1:**
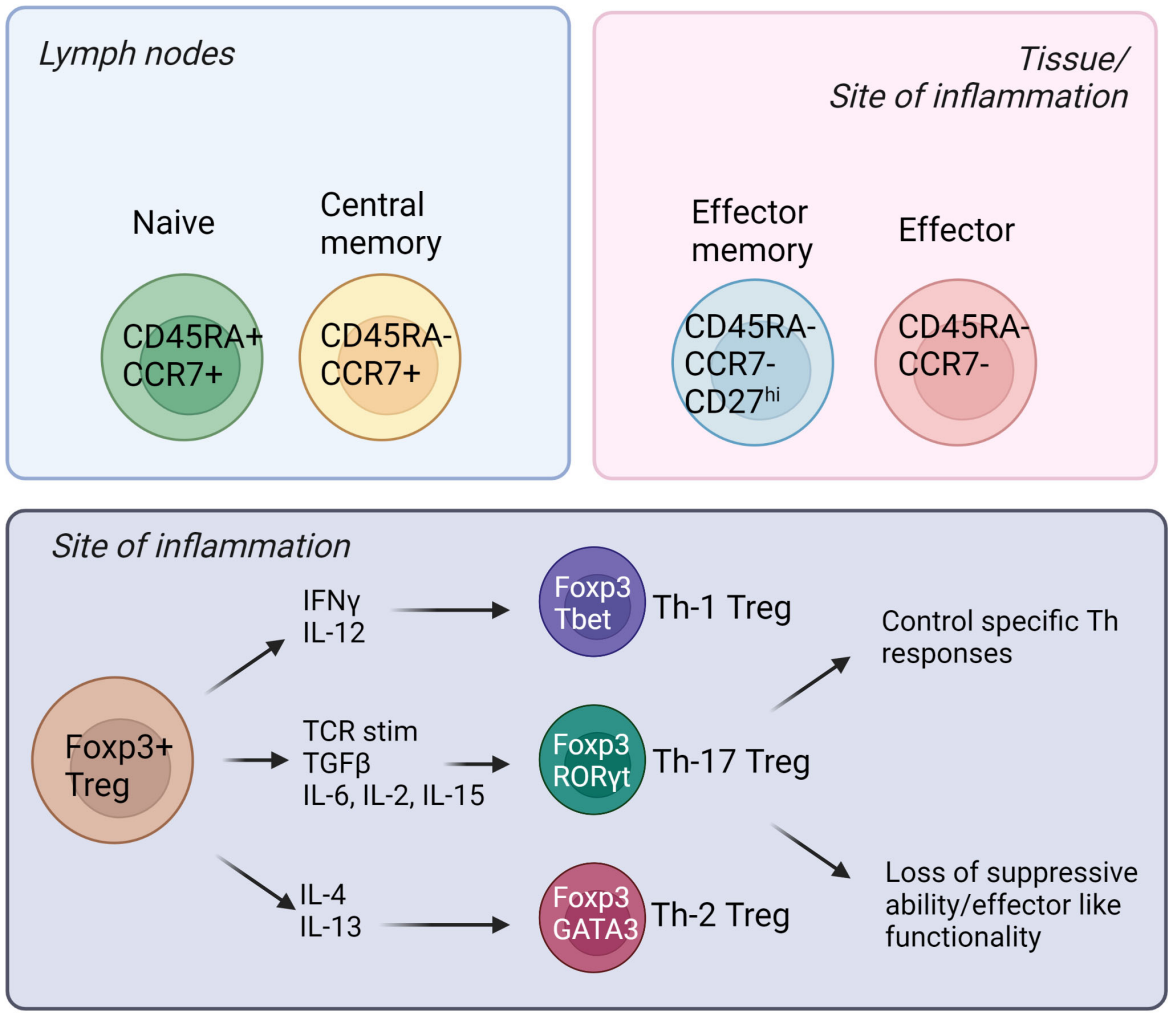
Different subsets of human Tregs in the lymph nodes, tissues, and sites of inflammation. Tregs emerge from the thymus and migrate into secondary lymphoid organs. Naïve and central memory Tregs reside in the lymph nodes while effector memory and effector Tregs can be found in the tissues and sites of inflammation. (Created by BioRender.com).

Natural Tregs (nTregs) emerge from the thymus, and they express the master transcription factor Foxp3 (forkhead box P3) (6). The most studied Treg population is CD4+; however there have been reports of CD8+ Tregs in the recent years (7, 8). In addition, there is a suppressive T cell population named Tr1 cells that are CD4+ Foxp3- and these cells express the co-inhibitory marker LAG3, CD49b and the inhibitory cytokine IL-10 (9). Tregs can also be induced from the naïve T effector cell populations (iTregs) and these cells have shown to be important in tissues such as the gut (10). Like Teffs, Tregs are heterogenous and different subpopulations exist—naïve/resting, central memory, effector memory and effector Tregs (11). The subpopulations are defined by differential expression of surface receptors such as CCR7, CD45RA, CD45RO and CD27 expression in humans; and CD62L and CD44 expression in mice (12, 13). Additionally, specific chemokine receptors and adhesion molecules are expressed, especially in tissue-directed and tissue-resident Tregs (14). For example, CCR4 is important for migration to skin and CCR9 is needed for homing to intestine (15, 16). Furthermore, memory Tregs can be divided into Th1, Th17 and Th2 Tregs, and these subpopulations emerge for two main reasons (17–19). Firstly, Tregs may differentiate into T helper-like phenotypes in order to adapt to the microenvironment, and better control Teff immune responses. Secondly, in certain inflammatory settings, Tregs lose their suppressive capabilities, and secrete pro-inflammatory cytokines (17–19). **Figure 1** summarizes some of these different subtypes of human Tregs and their associated markers.

Tregs exert their suppressive role in different ways including cell-cell contact, secretion of inhibitory cytokines, cytolysis, metabolic disruption, and modulation of DC maturation. Tregs can suppress the immune system in an antigen-specific way but also through infectious tolerance mechanisms (20). A well balanced Treg function is key for a healthy immune system. Defects in Treg homeostasis and function leads to autoimmunity and chronic inflammation while increased number and function of Tregs can aid in the establishment and spread of cancer (21). Tregs can be activated through various axes, including antigen recognition, cytokines and in recent years checkpoint molecules have been widely studied as important pathways which can regulate Treg development and function (22, 23).

**Figure 2:**
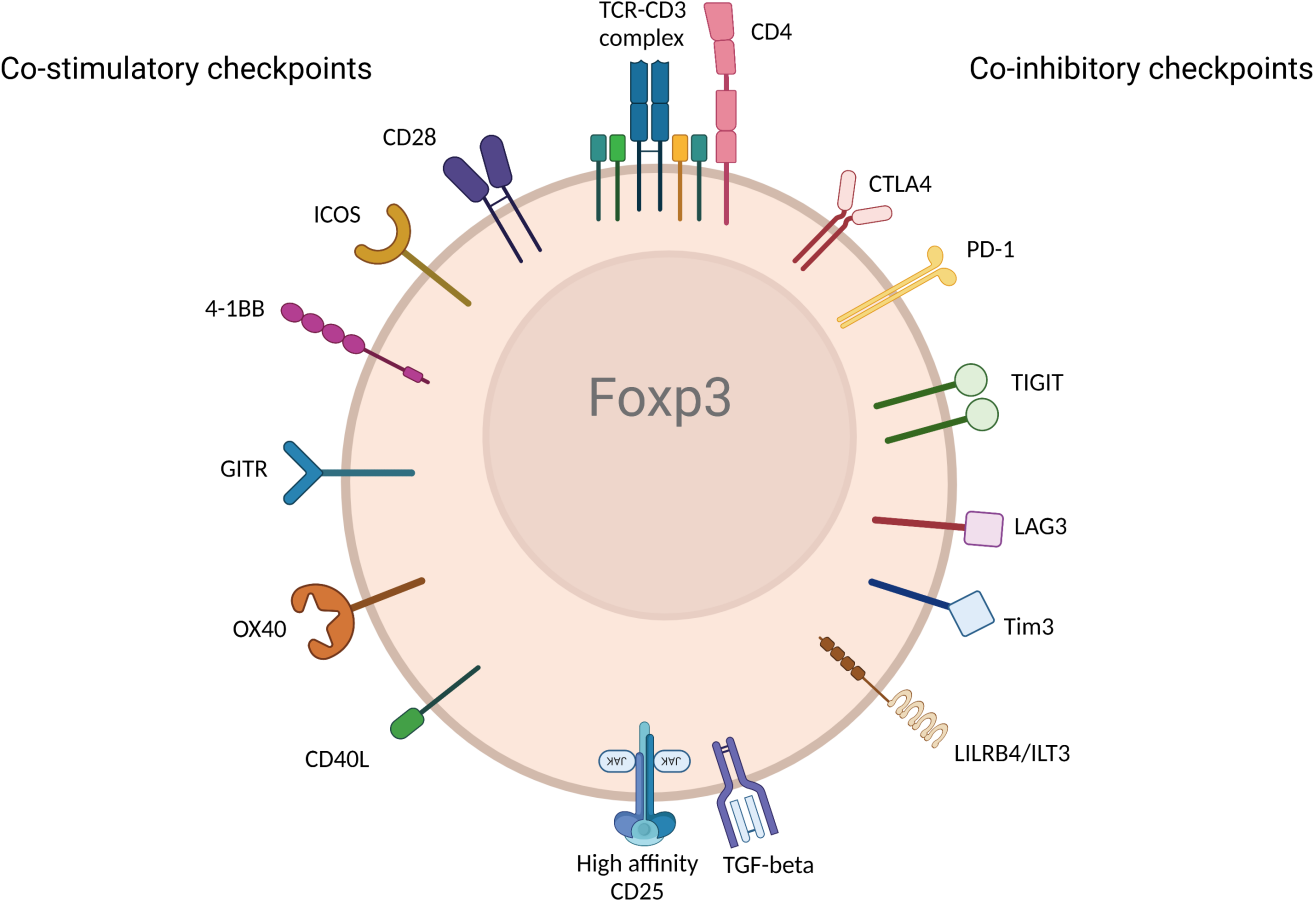
Immune checkpoint receptors expressed by Tregs. Various immune checkpoint receptors deliver unique signals to Tregs following engagement with their ligands expressed on APC and/or cells in tissue or diseased cells in different disease environments. The figure represents the most described co-stimulatory (left) and co-inhibitory checkpoints. (Created with BioRender.com).

Activation and regulation of T cells including Tregs require dual signals. The first signal (referred to as signal 1) is delivered when the T cell receptor (TCR) recognizes antigen in the context of Major Histocompatibility complex (MHC) and antigen on the antigen presenting cell (APC). The second signal (referred to as signal 2) is engaged when CD28—the major co-stimulatory molecule engages CD80 and CD86 on the APC (further discussed in detail below). Without either of the two signals, T cells and Tregs cannot be activated (24). Notably, the initiation and activation of T cells are further regulated by other checkpoint pathways. The immune response is fundamentally shaped and modulated by co-stimulatory and co-inhibitory receptors and their corresponding ligands and the balance between pro and anti-inflammatory signaling is maintained during homeostasis (25). In tumorigenic environments, cancer cells recruit Tregs, induce T cell tolerance and/or anergy, and stimulate inhibitory immune checkpoints (26). Inversely, in autoimmune settings, inflammatory molecules and cytokines activate stimulatory checkpoint pathways. Stimulatory checkpoint molecules are composed of two major families; CD28 superfamily composed of CD28 and ICOS; and Tumor Necrosis Factor receptor superfamily containing CD40, OX40, GITR, CD137 and CD27 (25, 27). Inhibitory checkpoint molecules are composed of a much larger number of described molecules such as CTLA-4, PD-1, TIGIT, LAG3, TIM3, LILRB4, VISTA, KIR, 2B4 and many others (28) (**Figure 2**). Deeper understanding of Tregs and different immune checkpoint pathways shaped the strong interest in targeting them as potential therapeutics in autoimmunity, transplantation, chronic allergic and inflammatory diseases (29, 30). Moreover, the finding that Tregs are present at tumor sites also raised the importance of targeting them to promote anti-tumor immunity (31). Therefore, all fields converged to the same objective to selectively manipulate Tregs to inhibit or promote their function in disease. While there is rich literature on the role of immune checkpoint pathways in modulating Treg homeostasis and function, there remains some controversies and outstanding questions. Comprehensive understanding of the impact of co-stimulatory and co-inhibitory pathways on Treg modulation will empower next generation therapeutics such as biologics, small molecules and Chimeric Antigen Receptor (CAR) cell therapy approaches. In this review, we discuss the biological impact of different checkpoints pathways, provide perspective from mouse and human studies, and share some insights about the modulation of these pathways in the clinic.

## The role of co-stimulatory pathways in Treg homeostasis and function

### CD28

CD28 is (44kDa, type I transmembrane protein) expressed on the surface of the majority of naïve CD4 and CD8 T cells as a glycosylated, disulfide-linked homodimer, and consists of a single extracellular Ig-V-like domain. CD28 was first characterized by a monoclonal antibody generated by John Hansen and Paul Martin in 1980 (32). They demonstrated that CD28 is a differentiation antigen expressed on thymocytes and majority of mature T cells. Cloning and expression of CD28 unveiled that each CD28 monomer contains 134 extracellular amino acids with a single transmembrane domain and a short cytoplasmic tail with no enzymatic activity. Each of the four exons contained in the CD28 gene defines a functional domain of the molecule (33, 34). CD28 binds both ligands CD80 (B7 or B7-1) and CD86 (B7-2). Additionally, CD28 can bind ICOS (35). Furthermore, cytotoxic T lymphocyte-associated protein-4 (CTLA-4) can oppose CD28, competes for binding the same ligands on APCs and induces CD28 downregulation *via* endocytosis (36). CD28 can generate a costimulatory signal (signal 2) once the TCR is engaged but is unable to activate T cells independently. In the absence of TCR stimulation, CD28 binds to CD80 monovalently and this weak interaction doesn’t generate a strong signal. However, in the presence of TCR activation, the homodimer form of CD28 binds to CD80 bivalently. Upon co-ligation, CD28 helps amplify the TCR signal in T cells. CD28 engagement on T cells induce the formation of distinct molecular patterns. It also induces cytoskeletal rearrangements and formation of an immunological synapse (37). CD28 is constitutively expressed in 80% of CD4 and 50% of CD8 T cells in human while all mouse T cells express CD28. Loss of CD28 expression is observed mainly in the CD8 T cells and was reported in human during aging and autoimmune disease (38). However, its expression is never downregulated in mice (39). The co-stimulatory function of CD28 has been utilized as a therapeutic factor in immunotherapy in oncology, especially in Chimeric Antigen Receptor (CAR) cell therapies and biologics (40, 41). However, it has also been an intricate balance to fully unleash the activating potential of CD28, since overstimulation can lead to cytokine storm and toxicity. Some of these CD28 activating therapies are discussed later in the review.

Like all other T cells, co-stimulatory signals are required for Tregs to become fully functional. Early studies have yielded conflicting results on the role of CD28 signaling in Tregs. This first became apparent in mice with the observation that spontaneous diabetes is exacerbated and the number of CD4+CD25+ Tregs is decreased in both CD28-deficient and B7-1/B7-2-deficient NOD mice (42). Tai et al. unveiled the important role of CD28 co-stimulation in thymic Treg differentiation. They reported that CD28 knock out mice had fewer Tregs in the thymus and these cells were unable to suppress the proliferative response of T cells *in vitro* (43). Likewise, Liang et al., showed that CD4+CD25- cells can be converted into Tregs *in vivo* through a B7-dependent pathway in a thymic independent manner. Later using CD28 knock out T cells, Guo et al. attributed this thymic independent peripheral Treg generation process to CD28 rather than CTLA-4 ligation of B7 both *in vitro* and *in vivo* (44–47). The role of CD28 in Treg function was further confirmed by Zhang et al. They developed CD28-deficient Treg mice in which CD28 is deleted under control of the FOXP3 promoter. While Treg numbers were preserved in these mice, these cells had lower levels of CTLA-4, PD-1 and CCR6 and they developed a systemic autoimmune condition affecting mainly the liver and the skin. This result was consistent with loss of suppressive function in CD28-deficient Tregs and failed to maintain immune surveillance *in vivo* (48). Other studies confirmed the stipulation of CD28 for *in vitro* Treg generation, as enriched Treg populations can only maintain their Treg phenotype and suppressive activity when co-stimulated with anti-CD28, even when expanded in the presence of rapamycin (49). To further assess the role of CD28 in Treg maturation and function, Zhang et al. performed a detailed analysis of manifestation of skin disease in CD28 Treg deficient mice. They demonstrated that CD28 activation is essential for optimal maturation of CD44loCD62Lhi central Tregs (cTregs or naïve Tregs in the secondary lymphoid organs) into CD44hiCD62Llo effector Tregs (eTregs) and induction of CCR6 in the latter cells. While CD28-deficient Tregs had normal function when injected directly to the skin, they were unable to home to inflamed skin due to the downregulation of CCR6 which is required for tissue homing (25).

Hombach et al. highlighted the role of CD28 co-stimulation in human Treg cells by comparing the activation requirement of resting human CD4+CD25+ Tregs to CD4+CD25- T cells. They demonstrated that the stimulatory conditions that induce T cell proliferation are not sufficient to induce Treg proliferation since Tregs require a combination of an intense TCR signaling with a very strong CD28 co-stimulation. Despite high levels of CD25 expression in Tregs, high concentrations of IL-2 could not substitute CD28 signaling (50). With the expansion of immunotherapy and the use of Treg as living therapies for autoimmune conditions, there has been great interest in generating high quality Tregs. He et al. demonstrated that a single CD28 stimulation of FACS sorted nTregs induces proliferation, high levels of Foxp3 expression, reduces pro-inflammatory cytokine production potential, preserves the TSDR demethylation and maintains high suppressive capacity (51).

**Figure 3:**
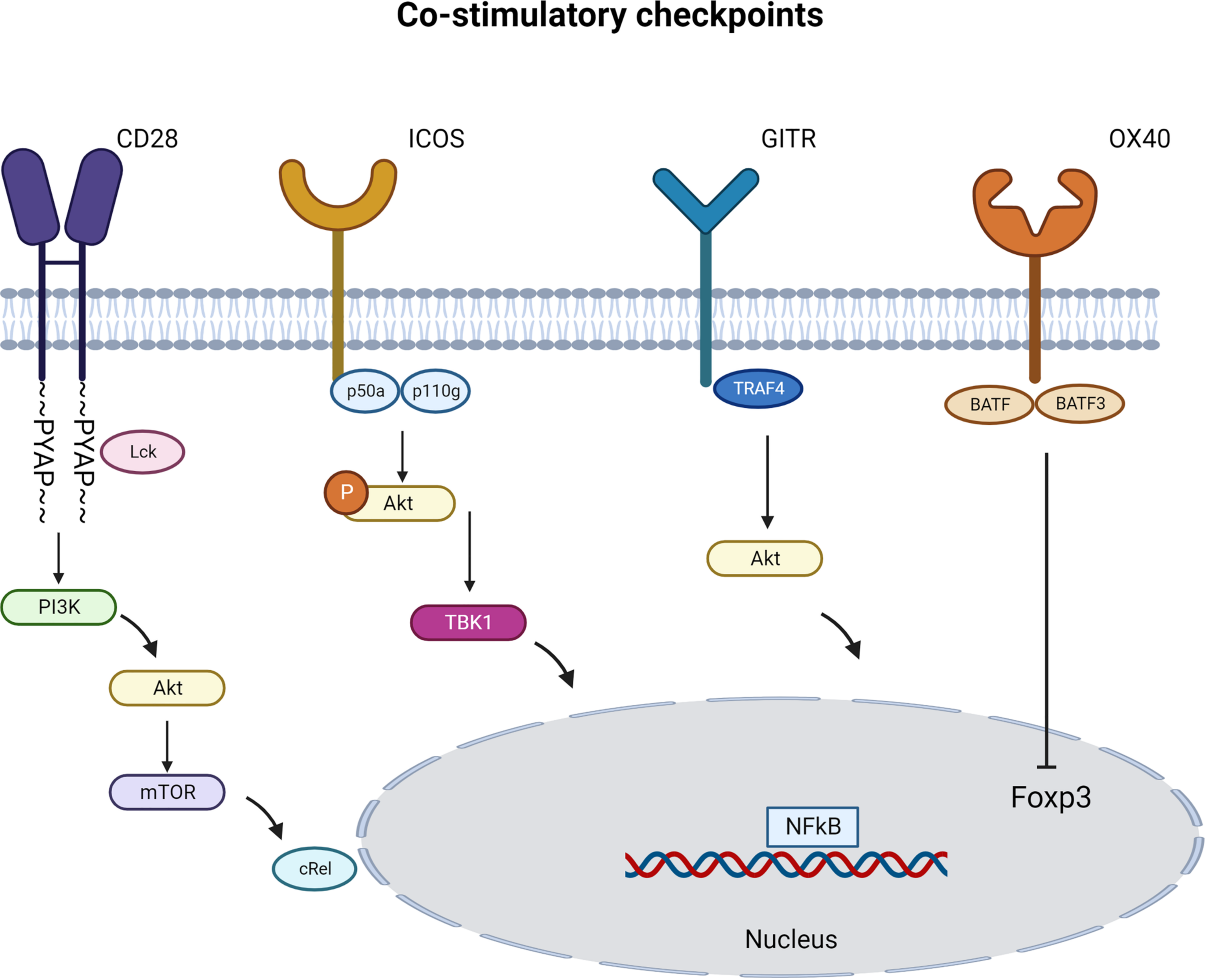
Immune Co-stimulatory signaling pathways specific to Tregs. Immune co-stimulatory checkpoints signaling are described in different cell types but only some are detailed in Tregs. The figure represents the most studied pathways. (Created with BioRender.com).

CD28 engagement activates several cytosolic signaling pathways. Using CD28 transgenic mice, several groups uncovered different signaling pathways of CD28 that are distinct in Tregs and T cells. Tai et al. proved the important role of the PYAP motif within the cytoplasmic domain of CD28 that require an intact Lck-binding motif. This CD28 cytosolic tail interaction with Lck is required both for efficient Treg generation and for IL-2 production. Similarly, CD28 activation in CD4+CD25- T cells lead to STAT3 Tyr^705^ phosphorylation in an Lck-dependent manner to drive Foxp3 expression (52). Vang et al. demonstrated that CD28 drives Treg differentiation in the thymus *via* the PYAP motif through a subsequent activation of the proto-oncogene c-Rel and Nuclear factor kappa B (NFkB). Engagement of c-Rel induces high-level expression of the IL-2R complex on Treg progenitors which allow Tregs to respond to IL-2 (53). (**Figure 3**). Other studies pointed the role of Phosphoinositide 3-kinase (PI3K) pathway in Foxp3 transcription induction. Scotta et al. demonstrated that CD28 signals independent from TCR, results in PI3K/AKT pathway activation and this is sufficient to induce the transcription of FOXP3 in a small proportion of primary CD4+CD25- T cells committed to express Foxp3 (54). CD28 ligation was also sufficient to activate PI3K target protein kinase B (PKB; c-AKT) and inactivate phosphorylation of PKB target glycogen synthase kinase-3 (GSK-3) (55). These results were complemented using CD28 superagonist in FACS sorted Tregs in the presence or absence of PI3K-inhibitor or mTOR-inhibitor. The Treg activation in the presence of exogenous rhIL-2 induced an increase in Foxp3 expression and a decrease in inflammatory cytokine production that was dependent on distal mTOR and proximal PI3K signaling (51). Collectively, these results suggest a key role for CD28 co-stimulation in promoting Treg function.

### ICOS (CD278)

ICOS (55-60kDa) is a disulfide-linked homodimeric T-cell surface glycoprotein. First identified in humans two decades ago, ICOS is the third member of the CD28 co-stimulatory family that is expressed on different populations of activated T cells comprising CD8+ cytotoxic T lymphocytes (CTL), CD4+ (Th1, Th2, Th17 and Tfh) and CD4+Foxp3+ Tregs (56). TCR engagement and/or CD28 co-stimulatory signaling induce increased expression of ICOS on CD4 and CD8 T cells. ICOS engagement by ICOS ligand (ICOSL) induces a wide range of cytokines and increases Treg suppressive function. Thus, distinct expression pattern of ICOSL can regulate ICOS ligation (57, 58).

During homeostasis, ICOS is constitutively expressed in approximately 20% of Foxp3+ Tregs, with the majority of ICOS+ Treg being CXCR3+ (Th1-like Tregs) or CXCR3-CCR6+CCR10- (Th17-like Tregs) (59). Despite substantial homology with the other family members, co-stimulatory CD28 and co-inhibitory CTLA-4, ICOS cannot bind the same receptor due to the lack of MYPPY motif (60). However, it binds its unique ligand (ICOSL/B7-H2) constitutively expressed on B cells, macrophages, DCs, and somatic cells (61, 62). ICOS can be induced by TNF-alpha on various non-lymphoid cells including endothelial cells (63), lung epithelial cells (64), mesenchymal stem cells (65), fibroblasts (66) and tumor cells (67). Likewise, ICOS is highly expressed on tonsillar T cells. These latter cells are imperative for germinal center formation and B-lymphocyte maturation (56). The crosslinking of ICOS and ICOSL has stimulating activities, promoting an anti-tumor response by Th1, CTL and T follicular helper (Tfh) and a pro-tumor response mediated by Tregs and Th2 cells in the tumor microenvironment. On the other hand, ICOS engagement by its ligand is essential for the generation, function and maintenance of Tfh cells that help germinal center formation and auto-antibody production in autoimmunity (25, 58, 68). In contrast to the CD28 pathway, ICOS co-stimulation results in inefficient IL-2 production by activated T cells. Nevertheless, other cytokines including IFN-γ, IL-4, IL-10 and IL-21 are frequently more efficiently produced (69, 70). Mechanistic studies by Chen et al., proved that ICOS signal plays a key role in regulating suppressive function and survival of Tregs. *In vitro* activation of CD3 and ICOS favor the interaction between nuclear factor of activated T cells (NFAT) and Foxp3 leading to upregulation of Foxp3 downstream regulatory genes. ICOS promoted anti-apoptotic activity through PI3K-AKT pathway increasing Treg survival and function (71–73).

ICOS engagement leads to the recruitment of both p50α for AKT activity (72, 74) and p85α (75) regulatory subunits of PI3K, in parallel to the recruitment of the p110δ catalytic subunit. Recently, the recruitment of the p85α subunit was linked to the differentiation of Tfh cells (72). Contrarily to CD28, ICOS induces a more robust PI3K signaling and a less robust MAPK signaling with a specific recruitment of TBK1 (58). Using an ICOS YF mice model that are incapable of activating PI3K signaling, O’Brien et al. demonstrated that PI3K-independent signaling downstream of ICOS plays a crucial role in Treg stability in the context of chronic inflammation (76). Activation of PI3K increases calcium mobilization triggered by the TCR. ICOS ligation induces a stronger AKT activation than CD28 engagement. The entire cytoplasmic domain of ICOS is necessary for its co-stimulation and calcium mobilization. This co-stimulatory function depends on the unique transmembrane domain (TMD) of ICOS, responsible for promoting association with the tyrosine kinase Lck. Transmembrane domain-enabled Lck association is essential for p85 recruitment to ICOS and consequent PI3K activation. Lck triggers both the bystander and co-stimulatory signaling activity of ICOS. Replacement of ICOS transmembrane domain, while keeping the cytoplasmic domain intact, fails to support Tfh development of germinal center formation *in vivo*. When the ICOS transmembrane domain was transplanted onto a CAR, it boosts interactions between T cells and antigen-presenting target cells (77). Therefore, the third generation of CAR T cell therapy comprising the ICOS in a transmembrane signaling demonstrated a superior efficacy and an increased persistence *in vivo* (78).

Abrogation of the ICOS pathway in NOD mice exacerbated the T1D disease pointing to the important role of this pathway in tolerance. In this model, ICOS expression discriminated Foxp3- T cells from Foxp3+ Tregs and specifically designated a subset of intra-islet Tregs with amplified potential to expand, produce IL-10 and mediate suppressive function both *in vitro* and *in vivo*. Blockade or genetic deficiency of ICOS abrogated Treg mediated protection from T1D and exacerbated the disease in BDC2.5-NOD mice (79). There is a strong core of evidence that ICOS+ Treg are more sensitive to IL-2 than their ICOS- counterparts. In a colitis model, ICOS deficiency resulted in an increased induction of IL-10 in CD4 T cells but reduced accumulation of Foxp3+ cells in large intestine. ICOS- Treg displayed increased methylation of Foxp3 conserved non-coding sequence 2 (CNS2) and preferentially downregulated Foxp3, which renders these cells unable of reverting the gut inflammation (80). In a model of hypersensitivity to 2,4-dinitrofluorobenzene, Vocanson et al. demonstrated the Hapten-specific Tregs expanding in response to their cognate antigen *in vivo* are mainly ICOS+ Treg, and by using reporter mice they demonstrated that these cells were derived from the expansion of natural Treg and were dependent on innate cells such as DCs (81). In Toxoplasma and Mycobacterium tuberculosis models, they described an expansion of Teffs and a loss of Treg frequency in the brain and lung of ICOS deficient mice respectively (76, 82).

In human melanoma patients treated with high dose IL-2 therapy, the ICOS+ Treg population was the most expanded and the most proliferative lymphocyte population in the blood. Melanoma patients with enhanced expansion of ICOS+ Treg in blood following treatment had unfavorable clinical outcomes than patients with fewer ICOS+ Tregs (83). For instance, ICOS^hi^ Tregs had superior immunosuppressive capacity compared to ICOS^lo^ Treg isolated from melanoma patients. These ICOS^hi^ Tregs were also able to induce suppressive IL-10 producing Tr1 cells from CD4+T cells (61). Similarly, a high infiltration of ICOS+ Treg in hepatocellular carcinoma indicated a worse prognosis (84). Moreover, in the tumor microenvironment, Tregs express increased levels of ICOS and Foxp3 and secrete higher levels of IL-10 and TGF-b (59). There is a clear understanding of the exacerbating role of ICOS+ Tregs in the disease in the immune-oncology field; however on the flip side, data are controversial in autoimmunity. In active SLE patients the ratio of ICOS+ Tregs to ICOS+ T responder CD4+ cells is significantly reduced and ICOS- Treg reduction is observed (85). Whereas in RA patients, ICOS+ Tregs were increased compared to normal controls, and such an increase was accentuated in patients with inactive RA compared to patients with active RA. Additionally for the patients with active RA the expression of suppressive cytokines (IL-10, TGF-β and IL-35) decreased while, expression of IL-17 increased compared with inactive RA, suggesting that ICOS+ Tregs may play an inflammatory and inhibitory function in a context-dependent manner (77).

### 4-1BB or TNFRSF9 (CD137)

TNFRSF9 also known as 4-1BB or CD137 is an inducible member of the tumor necrosis factor receptor (TNFR) family that plays the role of a co-stimulatory receptor. It was first discovered in 1989 on activated T lymphocytes (86, 87). It is expressed on activated CD4 and CD8 T cells but also on NK cells, B cells, neutrophils, dendritic cells, eosinophils, mast cells (88–91), endothelial cells, and some tumor cells (92–94). Tregs also expresses CD137 and its expression increases after activation (95–98). Whereas, its only known TNF-related ligand, 4-1BBL, is expressed by activated APCs (99, 100). When 4-1BB engagement by 4-1BBL is coupled with a strong TCR signaling, it provides co-stimulatory signals to T cells even in the absence of CD28 signaling. It enhances IL-2 and IFN-g production by Teffs and CTLs, respectively. Although, CD4 and CD8 T cells are both stimulated with 4-1BB *in vitro*, 4-1BBL/4-1BB crosslinking preferentially activates CD8 T cells (91, 101). There is also support that 4-1BB signaling in conventional T cells drives an excessive production of IL-2 responsible for escaping the Treg suppressive effect (102). Zheng et al., demonstrated that engagement of 4-1BB on thymic Tregs induces their proliferation both *in vitro* and *in vivo* while maintaining their suppressive ability despite the absence of detectable IL-2 indicating this pathway doesn’t involve IL-2 production (103). Similarly, data from Elpek et al. support the use of 4-1BBL as a means of proliferating Tregs *in vivo* (104). Likewise, 4-1BB is also able to downregulate the functions of Th cells, either by anergizing them or by enhancing the generation of Tregs (105–107). On the contrary, Akhmetzyanova et al. showed that 4-1BB stimulation converted Foxp3+ Tregs into cytotoxic killer cells that were able to contribute to antigen specific tumor rejection *in vivo* (108).

In a type 1 diabetes model, targeting 4-1BB with a monoclonal antibody suppressed the disease in NOD mice when the treatment is initiated before the development of auto-reactive T cells and is dependent on Treg induction (109). In a colitis model administration of anti-4-1BB agonistic antibody led to the reduction of incidence and severity of colitis. This effect was linked to a reduction of IL-2 expression by Th1 cells and an increase in Tregs (110, 111). Similarly, in an Experimental Autoimmune Encephalomyelitis (EAE) and Imiquimod-induced psoriasis-like dermatitis models, 4-1BB agonist was able to reduce disease by modulating Th17 versus Treg balance (112, 113). The generation of iTregs in the gut was attributed to the expression of retinal dehydrogenase (RALDH) by DC, an enzyme that promotes retinoic acid that aids differentiation of iTregs in the intestinal mucosa (114, 115). Another mechanism by which Treg engagement of CD137 tunes down Teff activation is by transferring and internalizing the CD137-CD137L complex formed between Tregs and APCs to Tregs *via* trygocytosis and depriving APC from their CD137L stimulating ability (107). As many of the members of the tumor necrosis factor superfamily (TNFRSF), 4-1BB ligation recruits TRAF adapter proteins, particularly that result in increased NFκB and MAP-kinase signaling (67, 116–119).

Despite the controversial function of 4-1BB in Tregs, agonistic anti-CD137 mAbs are being tested as therapies for cancer and autoimmune diseases. While, their mechanisms of action are different in different disease settings, they remain not fully studied and possible collateral effect on immune responses needs to be further investigated (120). Tregs with low 4-1BB expression was associated with enhanced overall survival in non-small cell lung cancer, confirming the role of 4-1BB in promoting suppressive function in tumor microenvironment (121). Also, using a genome-wide RNA-Seq analysis of multiple human cancer types and purified Tregs, Freeman et al. demonstrated that 4-1BB had an increased selectivity for human tumor Tregs (122). In autoimmunity, data support the role of 4-1BB in promoting Tregs (27). Altogether, 4-1BB can affect the biology of Treg cells and drive the crosstalk with other immunomodulatory cells.

### GITR or TNFRSF18 (CD357)

Glucocorticoid-induced tumor necrosis factor (TNF) receptor (GITR) is a member of the TNF receptor (TNFR) superfamily. GITR is a 228-amino acids type I transmembrane protein distinguished by three cysteine pseudorepeats in the extracellular domain. It was described for the first time in 1997 by Nocentini in DEX-treated murine T cell lines (123). GITR is constitutively expressed on CD25+CD4+ Tregs (97, 124). After activation, its expression is upregulated on all T cell subsets (125). GITR is also detected on various myeloid cells; which include monocytes, macrophages, DCs and MDSCs (126, 127). GITR is engaged by its unique ligand (GITRL) that is expressed on activated APCs and endothelial cells (128–130). GITR and GITRL expression is reported on numerous cell types and is not limited to hematopoietic cells. A moderate expression is observed on keratinocytes and osteoclast precursors, and elevated expression on endothelial cells stimulated with type I IFN (128). GITR engagement with GITRL rescues T cells from anti-CD3 induced apoptosis, preserves their activation, proliferation and cytokine production (131, 132). GITR has a distinctive role for CD8+ and CD4+ T cells. GITR signaling induces the expression of CD137 on CD8 memory T cells, it could also lower the threshold for CD28 signaling on CD8 T cells (133, 134). While, on Tregs, GITR is a critical receptor in the differentiation of thymic Tregs (tTregs), and expansion of both tTregs and peripheral Tregs (pTregs) (135, 136). GITR signaling is mediated by recruitment of TRAF2 and TRAF5 that induce NF-κB, resulting in the upregulation of Bcl-Xi, an anti-apoptotic protein on activated CD8 T cell (137). GITR engagement by GITRL induces IL-9 production by a subest of Th cells in a TRAF6 and NF-κB dependent manner, yielding enhanced tumor-specific CTLs response (138). However, in the context of Treg, GITR induces a TRAF4 mediated induction of NF-κB (139). To sum it up, Ephrem et al., demonstrated that the effect of GITR signaling is complex and depend on the activation state of the Tregs and Teffs as well as the physiologic environment of the host (140).

Mouse studies showed that GITR signaling abrogates the suppressive function of Tregs both *in vitro* and *in vivo* (97, 141). In contrast to data obtained in mice, however, the engagement of GITR by huGITRL did not abrogate Treg suppression and promoted their expansion (142–144). In mouse tumor models, treatment with anti-mouse GITR agonistic Ab, did not affect GITR expression in Tregs among Tumor Infiltrating Lymphocytes (TILs) that remained high. Nonetheless, non-Treg CD4 and CD8 TILs exhibited high GITR expression as well. Interestingly, the GITR expression on CD8 and CD4 TILs corroborated the anti-tumor effects of GITR treatment (145). In a mouse melanoma model GITR signaling destabilized Treg by reducing Foxp3 expression in intratumoral Tregs but not in circulating one which improved anti-tumor immunity (146). Similarly, targeting GITR in a glioblastoma model using an agonistic antibody promoted CD4 Treg differentiation into CD4 Teffs, alleviated Treg-mediated suppression of anti-tumor immune response, and induced potent anti-tumor effector cells (147). Recent evidence from murine tumor models suggests that anti-GITR antibodies selectively reprogrammed Tumor infiltrating-Tregs (TI-Tregs). These antibodies caused downregulation of Foxp3, Helios and IL-10, while increased the levels of IFN-g production from TI-Tregs. GITR targeting antibodies play a crucial role *via* in regulating Helios expression, as anti-GITR treatment phenocopies Helios genetic deletion in Tregs. Similar observation was made in the context of Myasthenia Gravis model where GITR directly regulated Helios expression in Tregs (148, 149).

On the flip side, blockade of GITR and GITRL interaction in autoimmunity alleviates disease severity. In NOD mice, an agonistic treatment with GITR accelerated disease onset significantly. The activity was not attributed to a decline in Tregs but rather an activation of diabetogenic T cells. The role of Tregs was confirmed when a similar observation was made is CD28-/- NOD mice which lack Tregs. Likewise, agonistic treatment with anti-GITR (DTA-1) during the induction phase of EAE significantly enhanced the level of clinical severity (150, 151). In contrast, blockade of GITR with a neutralizing antibody led to a significant protection from diabetes even at late-stage disease (151). Comparison of cells from GITR-/- to GITR +/+ showed that GITR-/- Tregs are more suppressive *in vitro* and GITR-/- mice are less susceptible to developing RA in a CIA disease model (152).

### OX40 (TNFRSF4, CD134)

OX40 (also called CD134 and TNFRSF4) is another co-stimulatory receptor of the TNFRSF of an approximately 50kD which is a type 1 transmembrane glycoprotein first described in 1987 (153, 154). OX-40 is a well-known Tcell activation marker. It is induced in activated CD4 and CD8 T cells, as well as in neutrophils, NK and NKTs (155, 156). OX40 is a late costimulatory receptor. Artificial engagement by OX40L induces proliferation, cytokine production, and T cell survival. This effect is partly induced by the expression of antiapoptotic molecules of the Bcl-2 family including Bcl-xL, Bcl-2 and Bfl-1 as well as survivin (157). However, it does not interfere with priming naïve T cells, as OX40-deficient T cells show normal differentiation to Teffs *via* TCR signaling but are not able to remain alive (158, 159). OX40 is also considered as a key marker of Tfh cells that can promote their generation and maintenance. OX40 synergizes with ICOS to maximize Tfh responses and formation and maintenance of germinal centers (160). In mouse Tregs, OX40 is constitutively expressed and the OX40 ligand (OX40L or CD252) is broadly expressed on activated APCs, including DCs, B cells, and macrophages, as well as non-hematopoietic cells, comprising endothelial cells and smooth muscle cells (161). Some studies show that when OX40 is engaged on Tregs, it not only inhibits their function and generation but can additionally, impede the generation, differentiation, and suppressive function of IL-10-producing CD4 type 1 Tregs significantly (162, 163). Peloso et al. demonstrated that engaging OX40 on Tregs by an OX40 agonist does not intrinsically impair their function but rather enhances their inflammatory cytokines production such as IFN-g, TNF-a and Granzyme B (164). In a different study by Piconese et al., they showed that mast cells counteracted the Treg function by ligating the OX40, and together with IL-6 production they induced a differentiation toward Th17 (165). On the other hand, OX40L expression on ILC2s induced tissue-restricted T cell co-stimulation that was crucial for Th2 and Treg responses in the lung and adipose tissue. Additionally, the IL-33 administration resulted in an organ specific expression of OX40L on ILC2 with a concomitant expansion of Tregs (166).

In T cells, OX40 activates both PI3K and NF-κB pathways by forming a signalosome containing TRAF2, IKKa, IKKb, PI3k and AKT (157). However, in Treg precursors, OX40 co-stimulatory activates BATF3 and BATF that induce the production of a closed chromatin to repress Foxp3 expression in Sirt1/7-dependent manner. Additionally, OX40 can also activate PI3K-AKT-mTOR pathway and inhibit Foxp3 induction by phosphorylation and nuclear exclusion of the transcription factor FoxO (46, 167). Much of the signaling work for OX-40 has been done in Teffs and Tfh cells, and further detailed studies for signaling in Tregs would be beneficial.

Numerous tumor mouse models and preclinical studies investigated OX40 agonistic signaling as an anti-tumor therapy through TIL regulation. The anti-tumor effects of OX40 agonist antibodies has been reported in several mouse models and human preclinical studies (168). Considering the constitutive expression of OX40 in Tregs, OX40 signaling dominantly affects Tregs. However, OX40 stimulation has numerous effects that lead to tumor elimination. OX40 engagement not only mediated Treg inhibition that unleashed nearby DCs allowing the induction of an adaptive immune response, but concomitantly delivered a fitness signal to activated T cells (164, 165). In autoimmunity, inhibition of OX40L ameliorated CIA disease scores in mice even when the signal on OX40 activated cells is intact (169). Contrarily, Griseri et al. demonstrated that OX40 is indispensable for Tregs accumulation in the colon and it plays a crucial role in Treg mediated suppression of colitis. OX40 deficient Tregs underwent enhanced activation induced death which corroborated the importance of OX40 in delivering the survival signal after activation. This could be explained by the importance of OX40 in Tregs that allows them to compete with colitogenic Th1 OX40+ cells for interaction with OX40L in DC (170). In a mouse model of pemphigus, Iriki et al. showed that OX40+ Tregs played a essential role in constraining OX40 signaling in autoreactive T cells. Likewise, Tregs signaling through OX40L on DCs suppressed the expression of OX40L itself (171). In a GVHD model, blocking OX40 reduced infiltration of human T cells to target organs. It also decreased IL-21 and TNF producing T cells while promoting Treg responses without compromising the cytolytic activity of CD8 T cells (172).

In active SLE patients, Treg dysfunction is mediated by APCs in an OX40L-dependent manner. In active skin lesions, Tregs and OX40L expression colocalized. Ligation of OX40 by OX40L resulted in downregulation of Foxp3 in Tregs (173). The data is conflicting regarding the effect of OX40 agonist on Tregs with some studies demonstrating that OX40 blocks the suppressive function of Tregs, while others showing enhanced Treg proliferation (46, 164, 165). Many studies in the autoimmunity setting demonstrated consistently that OX40 limits Treg function (169, 172–175). Altogether, these results indicate that OX40 signaling may regulate Tregs in several ways, depending on numerous factors, such as cytokines and other stimulation conditions (70).

### CD40L (CD154)

CD40L or CD154 is the ligand for CD40. It is a 33-39kD type II membrane glycoprotein (176, 177). CD154 was first reported in 1992 by Lederman and colleagues (178). It was described as an activation-induced surface T cell marker that is involved in mediating contact-dependent Teff function. It engages CD40 on B cells driving B cell activation, maturation, and germinal center formation. But it is also essential for the final differentiation of CD4 T cells and the selection of TCR clonotypes during a T-dependent humoral immune response. Recent data demonstrated that CD154 is critical for the selection of T-cell clones throughout the negative selection exercise in the thymus (179). CD154 expression has been described in a variety of other cells, including platelets, mast cells, macrophages, basophils, NK cells, B lymphocytes, and non-haematopoietic cells (180). Increased expression of CD154 on T cells has been reported in different autoimmune patients (181, 182). Naïve Tregs express basal levels of CD154 that are upregulated upon TCR-triggered activation (183). Several studies demonstrated that expression of CD40 on DCs is essential to induce T cell tolerance and Treg accumulation. This signaling in IL-10-differentiated DC-10 contributes importantly to the expression of IL-10 potent activator and this is a modulator of Treg activation and function (183–185). Other studies pointed the important role of CD40-activated B cells in inducing and expanding Tregs from naïve precursors (186, 187). The abrogation of the CD40-CD40L interaction hinders the homeostasis of thymic resident Tregs by changing the levels of IL-2 but does not affect their precursor development (188). Nicole et al. demonstrated that CD154+ nTreg could be efficiently expanded by specific antigenic activation while preserving their suppressive activity (189, 190). In a HIV chronic infection model, soluble CD154 induced immunosuppression by expanding Tregs (191). Similarly, CD40 upregulation on mature DCs increased the Th17/Treg ratio *in vitro* during the pathogenesis of periodontitis in young patients (192). The blockade of CD154 was able to modulate this ratio and prolonged the survival of allogeneic corneal grafts in mice (193). In an influenza model, Ballesteros-Tato et al. showed that Teffs and Tregs compete for CD40 ligation on DCs, and CD4+ T cells are only required for robust influenza specific CD8 response when Tregs are present (194). Ferrer et al. extensively studied the effect of CD154 blockade in extending graft survival. They demonstrated that CD154 blockade reduced antigen-specific CD4+ T-cell accumulation and promoted migration of Foxp3+ Tregs to the graft. They also showed that anti-CD154 promotes conversion of donor reactive CD4+ T cells into CD25+ Foxp3+ induced Tregs (195). Using a DEREG mouse model Lee et al. demonstrated that a short-term blockade of CD154 could lead to Treg mediated immune tolerance in the intrahepatic murine allogeneic islet transplantation model (196). Animal studies are supported by clinical data where CD154 blockade in relapsing-remitting multiple sclerosis patient demonstrated an increase of CD25+ T cells and anti-inflammatory cytokines (197).

## The role of co-inhibitory pathways in Treg homeostasis and function

### CTLA-4

Cytotoxic T lymphocyte antigen-4 (CTLA-4) or CD152, is a critical regulator of T cell responses. It was first described by Brunet et al. in 1987 in mice. It encodes 223 amino acid protein and was classified as a member of the Ig superfamily (198). Highly homologous to CD28, CTLA-4 binds to their shared ligands, CD80 and CD86, with greater affinity thereby preventing CD28 costimulation of T cells (199). Engagement of CTLA-4 also initiates signaling events, including recruitment of phosphatases and activation of ubiquitin ligases, which inhibit T cell activation (200–202). Unlike CD28, CTLA-4 has rapid and constitutive endocytosis process (203). While its expression is upregulated rapidly on conventional T cells following TCR engagement (204), CTLA-4 is transcriptionally regulated by Foxp3 (205) and therefore constitutively expressed on CD4+ Foxp3+ Tregs (206). Mice deficient in CTLA-4 develop lethal multi-organ autoimmune disease driven by CD4+ T cells (207–209). Disease also develops in mice with a Treg-specific depletion of CTLA-4, suggesting CTLA-4 plays a critical role in Treg biology (210). Evaluation of Tregs from these mice demonstrated that Tregs develop and survive in the absence CTLA-4 but are unable to suppress the activation of conventional CD4+ T cells. The dysregulated T cell response that developed when CTLA-4 was deficient on Tregs was associated with ineffective downregulation of CD80 and CD86 on APCs (210). Biochemical analyses have demonstrated that an interaction between CTLA-4 and the protein kinase C in Tregs is required for reduced CD86 expression by APCs (211).

There are numerous *in vivo* animal models that support a role for CTLA-4 in Treg function (212–214); however *in vitro* systems have yielded conflicting findings. Some reports have suggested that the suppressive capacity of human Tregs is largely dependent on CTLA-4, with CTLA-4 blocking antibodies reversing the inhibitory effect of Tregs on CD4+ CD25- T cell proliferation (215). Further, depletion of CD25+ cells also reduced the effect of CTLA-4 blocking antibodies on human T cell proliferation, suggesting that the impact of anti-CTLA-4 antibodies is in part mediated through Tregs (216). However, other *in vitro* studies revealed no impact of CTLA-4 blockade on the suppressive capacity of human Tregs (217). This could reflect variation in assay conditions as well as discrepancies between *in vitro* assays and complex *in vivo* systems. Indeed, there are reports that CTLA-4-deficient Tregs can retain functionality *in vitro* yet be unable to control inflammation *in vivo* (214). Additionally, conditional deletion of CTLA-4 in Tregs in adult mice was associated with protection from autoimmunity (218). Expression of the extracellular portion of CTLA-4 only by Tregs was sufficient to suppress the proliferative response of conventional T cells both *in vitro* and *in vivo*, while the intracellular portion of the protein was required to maintain TCR hypo-signaling in Tregs (219). Cumulatively, these data suggest that CTLA-4 can contribute to the suppressive capacity of Tregs, but its role may be context-dependent where the nature of the stimulus or the presence of compensatory suppressive mechanisms may influence the overall activity of Treg.

### PD-1

Programmed death 1 (PD-1) or CD279 is also a member of the Ig superfamily and is encoded by PDCD1 gene and composed of 288 amino acid residues (220, 221). PD-1 contains an intracellular immunoreceptor tyrosine-based inhibitory motif (ITIM) and serves as a central negative regulator of immune responses (222). It is expressed on the surfaces of activated T-cells, B-cells, dendritic cells, monocytes and natural killer cells (223–225). In 1989, Smith et al. described the phenomena of apoptosis or programmed cell death that the abnormal thymic T cells undergo in the thymus during cell maturation (226). This observation led to the discovery of the gene responsible for this programmed death by Honjo’s team in 1991 and elucidating its real function as a negative regulator of the immune responses of T cells (227). PD-1 can bind two ligands PD-L1 (228) and PD-L2 (229). PD-L1 is broadly expressed in inflamed tissues (221), while expression of PD-L2 is restricted to APCs (229). PD-L2 has three-fold higher affinity for PD-1 than PD-L1 (230). Mice deficient in PD-1 spontaneously develop autoimmunity, with the specific manifestations linked to the genetic strain on the animal, suggesting that in the absence of PD-1, the overall immune response is not differentially skewed but rather unleashed (231–234).

In addition to its broad expression pattern on a variety of activated immune cell populations, PD-1 as well as its ligand PD-L1, are expressed on the surface of Tregs. Using mice that selectively lack PD-1 in Tregs, Tan et al. demonstrated an improvement of the experimental autoimmune encephalomyelitis (EAE) and protection from diabetes in nonobese diabetic (NOD) mice (235). PD-1 deficient Tregs display an activated phenotype and an enhanced immunosuppressive function linked to a reduced signaling through the PI3K–AKT pathway (235). Likewise, the presence of PD-1 expressing Tregs in the tumor microenvironment is a signature of dysfunctional exhausted Tregs that have an increased IFN-g secretion and are unable to suppress the anti-tumor immune response (236). In the clinic, the use of PD-1 blockade led to an augmented proliferation and suppressive function of tumor infiltrating Tregs that resulted in a rapid cancer progression called hyper-progressive disease in 10% of advanced gastric cancer patients (237). Animal studies demonstrated that PD-1 signaling blocks the AKT/mTOR pathway and promotes of Foxp3 and the development of iTregs from naïve T cells (238). These observations were confirmed in a human system, where PDL-1 expressing cells were able to induce conversion of Th1 cells into Foxp3 expressing Tregs capable of conferring protection in a GvHD and colitis model (239, 240). Chronic stimulation leads to further upregulation of PD-1 levels on Tregs where, in settings of viral infection, its expression is critical for suppressing the anti-viral T cell response (241, 242). Additional exploration of human PD-1+ Tregs, including single-cell approaches, will be needed to better understand the consequence of PD-1 expression on Tregs in settings of infection, cancer and autoimmunity.

Tregs can directly inhibit Teff responses or can act on these cells indirectly by regulating APCs. Human Tregs have been shown to promote immune suppression by inducing upregulation of PD-L1 on DCs, with PD-L1 blockade reversing the Treg-induced suppressive activity of DCs (243). The ability of Tregs to directly inhibit B cell responses also involves the PD-1 pathway. Engagement of PD-1 on B cells by PD-L1/2 expressing Tregs directly inhibited activation, proliferation and antibody production from self-reactive B cells and promoted their apoptosis (244). Interestingly, the levels of expression of PD-1 are important for regulating Treg cell function as well. Performing a series of *in vitro* and *in vivo* testing of PD-1 pathway blockade, Wong et al. demonstrated that Tregs are functionally intact when the level of PD-1 expression is neither too high nor too low and that varied degrees of expression or engagement of PD-1 can trigger different immune responses (245).

### TIGIT

T cell immunoglobulin with ITIM domain (TIGIT) also designated as VSTM3, VSIG9 or WUCAM is a co-inhibitory receptor that was first described in 2009 expressed by T cells (246). Studies have confirmed its expression by other immune cells including B cells, NK cells, ILCs and pDCs (247–252). Later, studies demonstrated that TIGIT is highly expressed by Tregs (253–256). TIGIT is composed of an extracellular immunoglobulin variable domain, a type 1 transmembrane domain and a cytoplasmic tail with two inhibitory motifs and ITIM (immunoreceptor tyrosine-based inhibitory motif) and an ITT-like motif (257). TIGIT has multiple ligands including CD155, CD112, CD113, PVLR4 (258). However, TIGIT has the highest binding affinity to CD155, preventing the association between CD155 and the T cell costimulatory molecule CD226 (258, 259). Binding to either of the two ligands CD155 or CD112 on APCs prevents their maturation and confers a tolerogenic phenotype (246). Studies in mice have demonstrated that TIGIT expression serves as a marker for activated Tregs and Tregs expressing TIGIT have been demonstrated to be more potent suppressors than TIGIT negative Tregs (260). More specifically, TIGIT+ Tregs express elevated levels of markers including CTLA-4, CD25 and GITR as well as the transcription factor Foxp3. In addition, TIGIT+ Tregs produced higher amounts of effector molecules including IL-10, Fgl2 and granzyme B compared to TIGIT- Tregs. Ligation of TIGIT with an agonistic antibody directly induced production of both IL-10 and Fgl2 by Tregs *in vitro* and neutralization of Fgl2 *in vitro* reversed the enhanced suppressive capacity of TIGIT+ Tregs, demonstrating that Fgl2 is a key mechanism by which TIGIT drives enhanced Treg suppression (260).

TIGIT is highly expressed on human thymic-derived Tregs where its expression was associated with robust suppressive activity (254). TIGIT expression in combination with additional phenotypic markers can be used to define subsets of human Tregs. Fuhrman et al, for example, demonstrate that human Tregs which co-express both TIGIT and CD226 can produce cytokines such as IL-10 and effector genes, where TIGIT+ CD226- Tregs do not (254). Additionally, the combination of TIGIT and FCRL5 identifies a population of Helios+ Foxp3+ Tregs which do not produce pro-inflammatory cytokines and is highly enriched for suppressive activity (261). These studies highlight the heterogeneity that exists within the Treg lineage. Further evaluation of these specialized cell populations in different disease states and tissue compartments will be necessary to dissect the contribution of these various Treg populations to overall immune control and aid in our ability to better target these cell types for therapeutic benefit.

*In vitro* studies using natural ligand have provided insight into the mechanism by which TIGIT engagement impacts Treg signaling and function. TIGIT signaling leads to phosphorylation and nuclear localization of FoxO1 and suppression of AKT activity, ultimately preventing the reprogramming of human Tregs to pro-inflammatory Th1 Tregs by repressing IFNγ and T-bet expression (262). Studies in both mouse and human also suggest that TIGIT signaling may promote Treg stability. Agonism of TIGIT led to reduced expression of TCF7, a factor known to antagonize Foxp3 (254, 263). Further, signaling through TIGIT reduced the phosphorylation of both AKT and S6 and blocked the downregulation of Foxp3 in T cells stimulated through the TCR, supporting the idea that TIGIT plays a role in stabilizing the Treg lineage (264).

### LAG-3

Lymphocyte activation gene-3 (LAG-3) or CD223 is a 70kDa transmembrane glycoprotein. It is a member of the immunoglobulin superfamily and contains four extracellular Ig-like domains (265). LAG-3 is a CD4 ancestral homolog (265, 266). Similarly to CD4, LAG-3 binds MHC class II (MHCII) (267), additionally it was described to bind other receptors including fibrinogen-like protein 1 (FGL-1) (268), alpha-synuclein fibrils (alpha-syn) (269), galectin-3 (Gal-3) (270) and lymph node sinusoidal endothelial cell C-type lectin (LSECtin) (271). LAG-3 is another co-inhibitory receptor whose expression is transiently upregulated on effector cells following activation and more persistently expressed by Tregs, including thymic-derived Tregs, iTregs and Tr1 cells (272). While it is the most studied on T cells and Tregs, LAG-3 can also be expressed on unconventional T cells including gamma-delta T cells (273), mucosal-associated invariant T cells (274), invariant NKT cells (275), B cells (276, 277), plasmacytoid DC (278) and neurons (279).

There is evidence that LAG-3 can contribute to the regulatory capacity of Tregs through multiple mechanisms, including both cell-intrinsic and -extrinsic pathways. Antagonistic LAG-3 antibodies blunted the ability of both natural and iTregs to suppress the proliferation of Teffs *in vitro* and *in vivo* and ectopic expression of LAG-3 was sufficient to confer regulatory properties to T cells (272). Notably, the ability of ectopic LAG-3 to regulate T cell responses required expression of full-length LAG-3, with intracellular mutants not sufficient to confer suppressive activity. These data suggest that signaling through LAG-3 is required for T cell-intrinsic regulation. The intracellular pathways involved downstream of LAG-3 remain to be elucidated. Identification and characterization of the key signaling pathways regulated by LAG-3 will be critical for the development of therapeutics aimed at targeting LAG-3 activity.

Additional studies demonstrate that LAG-3 can also promote suppressive activity independently of its ability to deliver signals to the Treg. *In vitro* murine Treg/DC co-culture assays have shown that Tregs can inhibit antigen specific DC maturation in a LAG-3/MHCII-dependent manner, as LAG-3 deficient Tregs lost the ability to suppress upregulation of CD86 on DCs (280). Transwell studies demonstrated that the capacity of LAG-3 to block DC maturation was dependent on cell-cell contact, did not require the intracellular signaling domain of LAG-3 and involved a novel ITAM-mediated signaling event in DCs that involved the recruitment of SHP-1 (280). Cumulatively, these data highlight the potential for LAG-3 to induce inhibition in a bidirectional manner.

LAG-3 also represents a critical marker of Tr1 cells, a population of regulatory cells that produce high levels of IL-10 but do not express Foxp3 (281, 282). Forced expression of the transcription factor Egr-2 induced naive CD4+ T cells to express LAG-3 and IL-10 and to exhibit antigen-specific suppressive activity, suggesting a role for Egr-2 in LAG-3 regulation and Tr1 development (283). Anti-LAG-3 antagonist antibody treatment reversed the immunological tolerance induced by Tr1 cells in a mouse model of pancreatic islet transplantation supporting a role for LAG-3 in the activity of Tr1 cells, however, the precise role of LAG-3 in Tr1 function remains to be determined (284). Similarly, Jha et al., reported that blocking LAG-3 was associated with increased susceptibility to mercury-induced autoimmunity, and this response reduced Treg-mediated inhibition of DC maturation (285). Zhang et al. demonstrated that NOD mice lacking the cell surface expression of LAG-3 on Tregs exhibit delayed onset of Type 1 diabetes, and this was ascribed to the augmented Treg cell proliferation and activity (286). Altogether, these data suggest the important role of LAG-3 in modulating the Treg function and influencing the overall immune response in different disease settings.

### TIM-3

T cell immunoglobulin and mucin domain-containing protein-3 (TIM-3) also known as HAVCR2 is a member of the TIM family of immunoregulatory proteins. It was first described in 2002 by Monney and colleagues (287). TIM-3 has multiple ligands binding different epitopes on the TIM-3 extracellular V domain (288). The ligands include Galectin 9 which is a C type lectin (289), phosphatidylserine (PtdSer) (290), the glycoprotein CEACAM1 (291) and the alarmin high mobility group protein B1 (HMGB1) (292). TIM-3 has a unique feature because of the lack of known inhibitory signaling motifs in its cytoplasmic tail (288). It was originally described as a marker for exhausted effector T cells or IFN-γ producing CD4 and CD8 T cells (287, 288) but later described on a variety of immune cell types including myeloid cells (293), NK cells (294), mast cells (295) and Tregs (296). Subsets of Tregs have been described to express TIM-3, including some tissue resident Tregs. TIM-3 expressing Foxp3+ Tregs, in fact, make up minor fraction of the total Treg pool in a naïve mouse, but both the number and frequency of these cells increases significantly in both lymphoid and graft tissue during an allograft response (297). *In vitro* studies demonstrated that TIM-3 expression is upregulated on Tregs undergoing proliferation and its expression marks Tregs with enhanced suppressive capacity compared to TIM-3 negative Tregs. The enhanced suppressive capacity of TIM-3+ Tregs was associated with increased expression of CTLA-4, CD25 and IL-10 (297). Adoptive transfer studies revealed that while these cells have potent suppressive capacity, they did not prolong allograft survival as well as TIM-3 negative cells *in vivo* suggesting this represents a short-lived population of Tregs (297).

Oncology studies in both mouse and human support these observations, demonstrating an enrichment of TIM-3 expressing Tregs within the tumor microenvironment, with few of these cells observed in the blood or peripheral tissues (298, 299). In this setting as well, TIM-3 expressing Tregs demonstrate enhanced suppressive capacity and express higher levels of suppressive effector molecules than TIM-3 negative Tregs. Co-blockade of TIM-3 and PD-1 in preclinical tumor models was associated with downregulation of molecules that support the function of Tregs as well as an overall improvement in tumor clearance. Given the co-administration of TIM-3 and PD-1 blockade in these studies and the direct potential impact of these approaches on effector cells, additional studies are required to more specifically tease apart the impact of the TIM-3 pathway on Treg function.

### LILRB4

Leukocyte immunoglobulin like receptor B4 (LILRB4, also known as ILT3, LIR5, CD85K or HM18) is an ITIM-containing member of the LILR family of proteins. It was identified in 1997 as the homolog for the mouse gp49B1 inhibitory receptor (300). Several ligands have been reported to bind LILRB4 including CD166 (301), ApoE (302) and CNTFR (303). LILRB4 is broadly expressed across the myeloid lineage (including monocytes, macrophages, and DCs) as well as on some plasma cells (304). LILRB4 is highly expressed by tolerogenic DCs and thought to contribute to Treg induction by these cells (305). Expression of LILRB4 either on the surface of APCs or in soluble form, can engage with ligands expressed by activated T cells to drive T cell anergy and activation of Tregs (306). LILRB4 and LILRB2 expressing DCs support the conversion of alloreactive CD4^+^CD45RO^+^CD25^+^ T cells to Tregs (307). Some studies suggest that subsets of Tregs can themselves express LILRB4 (308). LILRB4+ Treg shows attenuated T cell receptor mediated signaling (308). LILRB4+ Tregs represent a small fraction of Foxp3+ cells in healthy peripheral blood but were found to be expanded in the circulation of allergic patients and in tumor infiltrating immune cells in various tumor models (309). Mouse studies demonstrate that the serine threonine kinase CK2 regulates the expression of LILRB4 in Tregs and highlights a role for this Treg subset in the control of Th2-cell driven inflammation. In the absence of CK2, LILRB4 expression was elevated on Tregs and this was associated with impaired Treg suppressive capacity and expansion of Th2-inducing dendritic cell populations (308). The authors hypothesize that LILRB4 upregulation on Tregs may represent a transient mechanism by which immune suppression is blocked when a productive immune response is required. These studies suggest that LILRB4 may play a complex role in Treg biology with the potential to either promote the induction of Tregs or to suppress Treg responses depending on the context and cell-type in which the pathway is engaged. As differences exist between murine and human LILRB4 both in terms of protein structure and expression, it will be important to demonstrate some of these mechanisms and explore the role of LILRB4+ Tregs in human assay systems or humanized mouse models.

## Clinical insights: Learnings from approved therapeutics and molecules in the clinic

The importance of checkpoint pathways in modulating different diseases have been highlighted with the presence of multiple approved therapeutics and many more in the clinical and pre-clinical development pipelines (85, 303, 310). Blocking and activating approaches with different modalities for different pathways have been pursued, and in 2018, James P. Allison and Tasuku Honjo was awarded the Nobel Prize in Medicine for the development of immune checkpoint blockade in cancer (311). In instances in which conflicting data is existent, this may be due to short-term versus long-term effects of treatment, different dosing strategies, and different patient cohorts. It may be even as simple as how the samples were collected, how the cells were isolated and how the samples were stored. Tregs are specifically sensitive to different storage conditions, and this can affect their viability. Below, we will discuss insights on the effect of these molecules on Treg biology.

### CD28 targeting therapeutics

Targeting CD28 with a superagonist antibody (TGN1412) in a first in human trial was a tragic failure, since the administered antibody induced a severe systemic inflammatory response and a cytokine storm in healthy volunteers (312). To overcome these adverse effects, this antibody was further engineered into TAB08 and tested clinically in healthy volunteers at very low doses and showed induction of IL-10 release in the circulation which is a signature of Treg activation. The positive results granted a phase Ib trial in RA patients followed by a double blinded phase II study to confirm the therapeutic effect in patients (313).

FR104 is a CD28 antagonist (a pegylated Fab antibody) and is currently being tested in Ph2 trials. In non-human primates, it has been demonstrated that this therapeutic is Treg sparring or Treg stimulating, yet data from further clinical studies are needed to confirm this finding (314). Acazicolcept (ALPN-101) is yet another experimental therapeutic in the clinic being tested by Alpine Immune Sciences in collaboration with Abbvie, and it simultaneously blocks CD28 and ICOS co-stimulation. Phase 1 healthy volunteer study demonstrated the safety, tolerability and PK/PD properties of this therapeutic, and currently being tested in a randomized, double-blind, placebo controlled Ph2 trials for SLE (315). It will be important to track the effect of ALP101 on Tregs among other things in these studies. Recently, Alpine Immune Sciences terminated enrollment in their NEON-2 study with Davoceticept (ALPN-202, CD28 co-stimulator, PD-L1 and CTLA-4 blocker) and pembroluzimab due to cardiogenic shock. It becomes more evident with every additional study that the modulation of immune cells (both effector and Treg) requires a sweet spot for therapeutic efficacy and safety and more precision medicine approaches will need to be utilized for future studies.

### CTLA-4 Ig therapy

As discussed above, CTLA-4 is constitutively expressed on Tregs and some of the suppressive capability of Tregs is mediated by CTLA-4 expression. Abatacept (CTLA-4 Ig fusion protein, Orencia™) was first approved in 2005 approved for Rheumatoid Arthritis, then later for Juvenile Idiopathic Arthritis (2008) and Psoriatic Arthritis (2017), Acute Graft versus Host Disease (2021) and it is being tested in the clinic for many other indications such as psoriasis, dermatomyositis, GvHD and others (316, 317). One study investigated the effects of Abatacept in RA patients and measured Treg numbers in the periphery at week 4 and 12 after treatment. 45 total RA patients with active disease were studied—30 received Abatacept and 15 received placebo treatment. The study showed that while Treg numbers were diminished in the periphery, the ex vivo functionality of Tregs were improved upon Abatacept treatment (318). In another study in juvenile idiopathic arthritis, Abatacept (10mg/kg i.v.) was administered to 10 patients (on day 0, 15 and 30 and monthly thereafter). Similarly in this setting, the number of peripheral Tregs was reduced at day 90 compared to normal controls (319). Bonelli et al., demonstrated that Abatacept treatment in RA patients increased proportions of Tregs but inhibited their suppressive capacity. This study also investigated the activation markers on Tregs such as CD69, CD71, HLA-DR and showed that activation markers were reduced after 2 and 4 weeks of therapy. There was also a reduction in CD95 and apoptosis mediated by this molecule (320). Diamanti et al., investigated the effect of Abatacept in anti-TNF therapy refractory patients (20 moderate to severe patients) and demonstrated that Treg frequency was not changed in the periphery. In this report, Abatacept treatment (10mg/kg) partially recovered the suppressive capability of Tregs after 6 months of treatment. One important point to note is that Tregs were defined as CD4+CD25+ cells in this study which is not the most robust way of identifying human Tregs (321). Szentpetery et al., investigated Treg numbers in the synovium and the psoriatic skin of 15 biologics-naïve psoriatic arthritis patients. Patients were randomized to receive i.v. Abatacept (10mg/kg) or placebo treatment. Treg (CD4+ Foxp3+) numbers in the synovium but not in the psoriatic skin of the patients were diminished with Abatacept treatment significantly improving clinical measures. It was interesting to see that Abatacept treatment was efficacious for joint-related outcomes but not for skin-related outcomes (322). These data points are good examples of how these co-stimulatory and co-inhibitory pathways can have tissue-specific effects. While tumor biopsy tissue has been more readily accessible for analysis of Treg modulation beyond the periphery, tissue data from autoimmune settings has been scarce. With the emergence of new technologies such as high dimensional immunofluorescence and spatial transcriptomics, our scientific understanding of tissue immunology and Treg function will deepen in the upcoming years.

Belatacept (Nulojix™) has 4 times higher affinity for CD86 and 2 times higher affinity for CD80 and is the newer generation of CTLA-4 blocker (323). In 2011, it was approved for prophylaxis of organ rejection in adults (324). In a small cohort study, Grimbert et al., studied the effect of Belatacept on mRNA levels of Foxp3 in biopsies from renal transplants and demonstrated that the Foxp3 levels were reduced only in the graft. This did not correlate with clinical efficacy since Belatacept treated patients had better functionality of graft after 1 year (325).

### CTLA-4 blockade

Anti-CTLA-4 blocking therapies have been approved and used for oncology indications mainly because they are able to remove the inhibitory breaks on effector cells. However, due to their constitutive CTLA-4 expression, Tregs are also a direct target of this therapy. Ipilimumab (Yervoy™), the first anti-CTLA-4 was first approved in 2011 and since then the indications for this medicine has been expanding along with combination therapy testing with other checkpoint inhibitors (326). One study by Sharma et al., demonstrated that the levels of Tregs were transiently increased 6 weeks after Ipilimumab therapy but returned to baseline by 3 months and stayed unchanged for 6 and 9 months in melanoma patients (10mg/kg iv every 3 weeks) (327). To mirror these results, a different study demonstrated the same outcome in melanoma patients that Tregs were transiently increased at week 6 but returned to normal after 3, 6 and 9 months and there was no difference in relapsed and relapse-free groups (328). Zhou et al., studied Ipilimumab treatment in patients with relapsed malignancy following allogeneic hematopoietic stem cell transplantation and demonstrated that the absolute counts of Tregs (CD4+CD25highFoxp3+) did not change during the study period (329). Kavanagh et al., studied a small cohort of progressive metastatic hormone-refractory prostate cancer patients in a Ph1 study. The subjects were dosed with escalating dose of Ipilimumab with a fixed dose of GM-CSF in a separate cohort. 3mg/kg anti-CTLA-4 only treatment resulted in increase of CD4+ Foxp3+ frequency and that Tregs maintained their level of both surface and intracellular reserves of CTLA-4 (330). One proposed mechanism of action is depletion of CTLA-4^hi^ cells, especially Tregs by ADCC in the tumor microenvironment. Specifically, some approaches are trying to generate second generation of anti-CTLA-4 therapies with deeper Treg-depleting capability such as with enhanced Fc binding. One group demonstrated that Fc-enhanced CTLA-4 antibodies had better efficacy in tumor bearing FcγR humanized mice due to their Treg depleting capabilities (331). Another study by Semmrich et al., generated proof of concept in mice that intratumorally administered viral vectors containing anti-CTLA-4 or anti-CTLA-4 in combination with GM-CSF was better than systemic administration of anti-CTLA-4. A follow up Ph1 study is ongoing with viral vector (anti-CTLA-4 and GMCSF) administration in patients with metastatic or advanced solid tumors (NCT04725331 (331)).

Tremelimumab is another anti-CTLA-4 fully human IgG2 antibody currently in development. There are multiple Phase 3 and Phase 2 studies ongoing worldwide (332). Comin-Anduix et al., studied the cell signaling events in PBMCs from melanoma patients that were treated with tremelimumab after TCR and cytokine receptor stimulation. Looking at CD4, CD8 T cells and monocytes, they demonstrated that pp38, pSTAT1 and pSTAT3 were increased, pLck, pERK1/2 and pSTAT5 levels were decreased (333). In a very small Phase 1 study, the effect of local radiation and tremelimumab treatment was studied in inoperable locally recurrent or metastatic breast cancer patients. The data demonstrated that one week post treatment, there was an increase in ki-67+ proliferating Tregs in 5 out of 6 patients (334). In a different study, Khan et al., demonstrated that Tremelimumab treatment increased proliferation of Teffs, secretion of IL-2 and IFN-γ however, did not change the proportion of Tregs while reducing their suppressive capacity (335).

### PD-1 and PD-L1 blockade

Nivolumab (Opdivo™) is a PD-1 antagonist and has been approved by FDA for the treatment of several different cancer types such as melanoma, non-small cell lung cancer, hepatocellular carcinoma, cervical cancer and head and neck squamous cell carcinoma (FDA: Hematology/oncology (cancer) approvals & safety notifications. 2019). One study studied the effect of Nivolumab in oral cavity squamous cell carcinoma in a Ph2 study with treatment on day 1, 14 and 28 (3mg/kg i.v.). Peripheral blood samples were collected before Nivolumab treatment and at the time of definitive oral resection. Progressive patients on day 28 proceeded with surgery while the patients improving received a fourth dose of Nivolumab on day 48. The findings demonstrated that while anti-PD-1 had opposing effects on CD4 (reduced) and CD8 (increased), it increased the proportion of Foxp3+ expressing cells in the periphery (336). In a different study, it was shown that treatment with Nivolumab pre-transplant of HSCT resulted in increased IFNg+ effector cells and potential rejection of the graft. Interestingly, posttransplant treatment of these patients with cyclophosphamide increased Tregs (337). To further build on this data set, Ikegawa et al., took a closer look into Treg numbers and phenotype in an exploratory study pre- and post-transplant with cyclophosphamide treatment. Their study demonstrated that cyclophosphamide treatment helps with the control of T effector cells and robust recovery of Tregs in the presence of pre-treatment with Nivolumab (338).

Another PD-1 antagonist is Pembrolizumab (Keytruda™, humanized IgG4k antibody) and its effects on different T cell populations have been studied. *In vitro*, Toor et al., demonstrated that treatment of PBMCs from primary cancer patients did not change Treg related markers such as CTLA-4, CD15s, LAP and ki-67. In this study, Pembrolizumab had the greatest reduction effect on PD-1 expression in CD4+CD25- cells compared to CD4+CD25+ T cells. As a follow up, the same group studied the effect of Pembrolizumab on iTreg generation *in vitro* and showed that pembrolizumab treatment inhibited iTreg generation and rendered Tregs less suppressive, especially at higher ratios. This was due to reduced IL-10 expression, mTOR and STAT1 activation and subsequent MAPK inhibition (339).

### Other immunomodulatory therapeutics

AMG557 (MEDI5872) is an anti-ICOSL antagonist and has been tested in the clinic in SLE, Lupus arthritis, Sjogren’s syndrome and Psoriasis. Single and multiple doses of AMG557 were tested in 112 SLE patients, and it was shown to be safe and tolerable with no change in the Treg frequency in the periphery (340). In a human clinical trial, OX40 agonist expanded non-Treg CD4 and CD8 T cells and upregulated OX40 expression on TIL Tregs (341, 342). GSK has developed an anti-LAG3 depleting mAb (ADCC enhanced, afucosylated) and this molecule has completed Ph1 testing in healthy volunteers and patients with plaque psoriasis. Overall, there was a reduction in LAG3+ cells; however no specific data on Tregs was reported.

## Concluding remarks

In this review, we provided a summary of our understanding of how immune checkpoints regulate Treg homeostasis and function. While our understanding has gotten deeper over the years, there is still much to explore. Some outstanding areas to investigate include interplay between different immune checkpoints and how they modulate Treg function, the role of immune checkpoint pathways in Treg and other immune and non-immune cell interactions, and the role of checkpoint pathways in the non-immunological functions of Tregs such as wound healing and repair. In conclusion, immune checkpoints play fundamental roles in controlling Treg biology and further evidence, especially from the clinic, will be necessary in order to further our understanding and build the next generation of therapeutics.

## Author contributions

MA, JK and SR all contributed to the writing and editing of the manuscript. All authors contributed to the article and approved the submitted version.
